# Acoustic Emission Signal of *Lactococcus lactis* before and after Inhibition with **NaN**_**3**_ and Infection with Bacteriophage c2

**DOI:** 10.1155/2013/257313

**Published:** 2013-11-17

**Authors:** Debasish Ghosh, John M. Stencel, Clair D. Hicks, Fred Payne, Didem Ozevin

**Affiliations:** ^1^Tribo Flow Separations, 2324 Lilac Park, Lexington, KY 40509, USA; ^2^Ferm Solutions Inc. 445 Roy Arnold Avenue, Danville, KY 40423, USA; ^3^Department of Animal & Food Sciences, University of Kentucky, Lexington, KY 40546, USA; ^4^Department of Biosystems and Agricultural Engineering, University of Kentucky, Lexington, KY 40546, USA; ^5^Department of Civil and Materials Engineering, University of Illinois at Chicago, Chicago, IL 60607-7023, USA

## Abstract

The detection of acoustic emission (AE) from *Lactococcus lactis*, *ssp lactis* is reported in which emission intensities are used to follow and define metabolic activity during growth in nutrient broths. Optical density (OD) data were also acquired during *L. lactis* growth at 32°C and provided insight into the timing of the AE signals relative to the lag, logarithmic, and stationary growth phases of the bacteria. The inclusion of a metabolic inhibitor, NaN_3_, into the nutrient broth eliminated bacteria metabolic activity according to the OD data, the absence of which was confirmed using AE data acquisition. The OD and AE data were also acquired before and after the addition of Bacteriophage c2 in *L. lactis* containing nutrient broths during the early or middle logarithmic phase; c2 phage m.o.i. (Multiplicity of infection) was varied to help differentiate whether the detected AE was from bacteria cells during lysis or from the c2 phage during genome injection into the cells. It is proposed that AE measurements using piezoelectric sensors are sensitive enough to detect bacteria at the amount near 10^4^ cfu/mL, to provide real time data on bacteria metabolic activity and to dynamically monitor phage infection of cells.

## 1. Introduction

Periodic, vibrational motion of *Saccharomyces cerevisiae* yeast cell walls was observed using atomic force microscopy (AFM) [1, 2]. The wall vibrations which were temperature dependent, had frequencies between 1.0–1.6 kHz and ceased after the addition of a metabolic inhibitor. The periodic vibrations were ascribed to forced, concerted cell wall motions caused by cellular metabolism and molecular motors such as kinesin, dynein, and myosin rather than to natural resonant cell wall oscillations. The cell wall amplitudes were 3 nm with forces ~10 nN; for human foreskin fibroblasts the cell wall motion from actin-myosin activity produced forces between 20–100 pN. Hence, the range of work ((force) × (amplitude)) associated with cell wall motion in eukaryotes can be estimated to be between ~20 × 10^−20^ J-to-30 × 10^−18^ J (2000 aJ-to-30 aJ). These values are well above the <10^−3^ aJ sensitivities known for commercially-acquired piezoelectric-based sensors.

The routine application of AFM techniques to study whether periodic motion of bacteria cell walls occurs during metabolism may be considerably more difficult than of yeast cell walls because bacteria have less rigid walls and are much smaller in size. Nevertheless, bacterial wall vibration as caused by molecular motors has been theoretically modeled to produce vibrational motion with frequencies up to 10 kHz [3]. To effectively monitor, such motion or the metabolic activity of bacteria in real time would offer significant benefits to microbiological investigations.

The observation of induced standing waves within the walls of algae Hydrodictyon, having a cell wall thickness resonant with the 1 MHz standing wave to which the algae was exposed, was first measured by Miller [4]. The natural vibrational motions of microbe cell walls have also been theoretically studied [5]. Although the modeling used a shell model with spherically-shaped cells like those for the subject bacteria, *Lactococcus Lactis*, ssp.* lactis*, discussed herein, and may not be as applicable to rod-shaped cells like those for *Escherichia coli*, and the natural oscillation frequencies of bacterial cells having radii between 0.5 *μ*m-to-30 *μ*m were calculated to range from 500 kHz-to-4.6 MHz.

Acoustic emission (AE) measurements have been applied to measure events as diverse as microcracking in metals, earthquake tremors, chemical reactions, and microbubble processes [6–9]. Detection of ultrasonic AE, produced during fluid flow through holes in mechanical systems [10], has also provided impetus for products that detect and pinpoint the location of tiny gas and liquid leaks from pipes. Although attempts were reported for acoustic detection of microbes using different model systems [11], the principles underlying the concept and its practicality in real time is still poorly understood, thereby requires further in-depth investigation. AFM data detecting the vibration of yeast cell walls caused by metabolic activity, the force associated with these motions, and the sensitivities needed to detect such vibrations suggested that piezoelectric sensors may provide an avenue to determine if AE is produced during bacterial metabolic activity and if cessation of the AE could be caused by the action of exposure to a metabolic inhibitor, or if AE was produced during infection of bacteria by a specific bacteriophage.

Hence, this paper reports on the application of piezoelectric-based sensors to determine whether AE is produced by *Lactococcus lactis *ssp.* lactis* C2 during growth in nutrient broths and whether changes in the AE accompany the exposure of *L. lactis* to a metabolic inhibitor and to a c2-specific Bacteriophage. *L. lactis* was chosen because it is nonpathogenic and produces lactic acid rather than gas during respiration. In other words, complications in data analyses may be decreased because of the absence of acoustic signals caused by the formation, coalescence, and bursting of gas bubbles from cell respiration.

## 2. Materials and Methods

An experimental apparatus was constructed to acquire high quality AE data from nutrient broths containing bacteria before and after exposure to metabolic inhibitors and bacteriophage. The apparatus contained two identical compartments in which bacteria growth cells were placed and from which AE was acquired using computer-controlled hardware and software. The compartments were sound insulated and vibration isolated to minimize the interference from acoustic sources external to them. The AE sensors and software used were manufactured by Physical Acoustics Corporation (PAC, Princeton Junction, NJ, USA). The sensors were model number R6 having sensitivity between 20–100 kHz. Acoustic signals were preamplified and then analyzed by using PAC AEWin and AEPost software.

An R6 sensor was mounted onto the inner wall of each compartment and used as a guard sensor, that is, guard sensors monitored AE from sources external to the bacteria growth cells. If coincidence occurred between signals from a guard sensor and the corresponding growth cell the software rejected the coincident signal from the growth cell data, thereby eliminating acoustic events from sources external to the growth cells.

**Figure 1:**
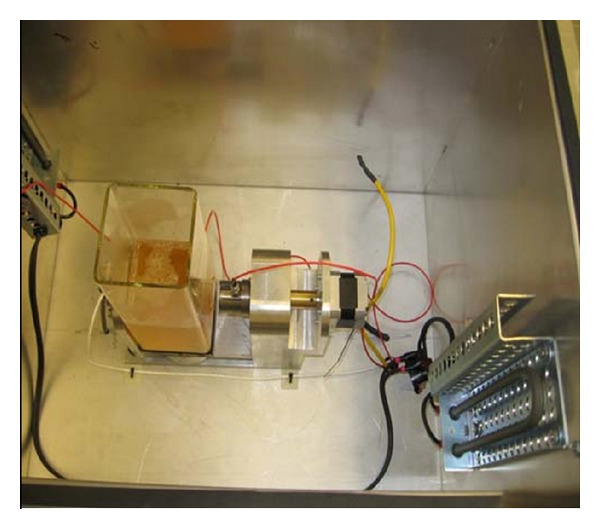
Picture of the inside of a sound chamber with AE sensors attached to the bacteria growth cell.

A picture of the inside of one of the compartments containing a square, bacteria growth cell, and AE sensor attachment mechanism is given in Figure 1. Firm and repeatable placement of up to two AE sensors on the walls of each cell was accomplished by using a computer-controlled stepping motor with feedback from a force sensor, mounted between the sensor back plate and the sensor holder, that gave direct readout values of the force of contact between the sensor faces and outside walls of the cells. A thin layer of silicon grease was applied to the face of each sensor before their placement into the sensor attachment mechanism. Underneath the cell support was a magnet attached to a stirring motor; it was activated intermittently to rotate a stir bar placed in each cell to cause mixing of the cell contents. Also shown in the figures are two heaters attached to the compartment walls; they were controlled using thermocouple readout and maintained a constant, optimum temperature of 32°C during the testing.

Each bacteria growth cell was large enough to contain 400 mL of nutrient broth. After cells were readied with nutrient broth, they were placed onto the cell mount within the compartments, the lids on the compartments were closed and then background AE data were acquired to establish threshold levels for each of the sensors. These levels were used as an input parameter to the software; if acoustic wave intensities above the threshold were detected the software enabled storage—each stored acoustic event is called a Hit in this paper. If intensities were below the threshold level the Hit was not stored. As a consequence, AE data presented herein were acquired using different base threshold levels, the outcome of which is different energy values for the detected emission.

All glassware used was sterilized at 121°C for 15 min before filling with Bacto M17 broth (Fisher Scientific Co., Pittsburgh, PA, USA) and with preprepared bacteria culture. Prior to use, the M17 growth medium was also sterilized at 121°C for 15 min and stored at 4°C. On the day of each experiment the medium was warmed to 32°C, poured into the glass cell which was placed into the AE test chamber, and then threshold emission levels were established.

The bacteria *Lactococcus lactis *ssp.* lactis* and the corresponding specific Bacteriophage c2 were obtained from the University of Kentucky Food Science −80°C culture library. *L. lactis* is gram-positive, usually spherical or ovoid in shape (0.5–1.2 *μ*m by 0.5–1.5 *μ*m), and occur in pairs and short chains. They are nonsporulating, nonmotile, and commonly used in the dairy industry for the manufacture of fermented dairy products like cheese. The by-product of *L. lactis* metabolism is lactic acid, which imparts a characteristic flavor in some cheese types.

One mL of frozen stock bacteria culture (−80°C) was added to a growth tube containing 10 mL of M17 broth and then incubated at 32°C for 16 h to produce a working bacteria culture. Then, 4 mL of the working or diluted culture was added to 400 mL of the medium contained in the square glass cells. For the tests to be described, the beginning bacterium amount in the cells was between 10^3^–10^7^ cfu/mL (cfu = colony forming units), and the phage amount was 10^9^ pfu/mL (plaque forming units).

After inoculating the bacteria, AE data collection was initiated. Collection was paused approximately every ~30 minutes to extract a 1 mL sample from each cell, on which optical density (OD) measurements were made using a Unico 2100 visible Spectrophotometer (600 nm wavelength, orange filter). During tests that lasted between 4–10 hours, the AE data were acquired continuously except when OD samples were extracted.

Besides “normal” growth of *L. lactis*, that is, growth during which the traditional lag, logarithmic (log), and stationary phases could be discerned by observation of OD patterns, and inoculated broths was also exposed to either a metabolic inhibitor or Bacteriophage c2. The inhibitor used was sodium azide (NaN_3_). Although there are a number of other inhibitors and biocides commonly used in basic and applied research, including the inhibitors sodium nitrite and molybdate and the biocides bronopol, formaldehyde, and glutaraldehyde, NaN_3_ was chosen because it is known to be an excellent reversible inhibitor of microbial respiration, and is highly soluble in water at the 0.05% concentrations used during the inhibition tests. The timing of exposing the bacteria-inoculated broths to Bacteriophage c2 was (a) during the early log phase (time ~100–150 minutes after bacteria inoculation); or (b) during the mid-log phase (time ~150–200) minutes after bacteria inoculation.

## 3. Results and Discussion

### 3.1. Bacteria Normal Growth

The AE data presented are the absolute energy of each acoustic Hit in attojoules (1 aJ = 10^−18^ joules). Because AE intensities were weak, an averaging of the energy-per-Hit over sequential one-to-five minute periods was accomplished from the beginning-to-end of each experiment. Five-minute averaging was accomplished first after each test was completed to establish vectors for the presence or absence of AE peaks. Then, one minute averaging was accomplished to provide more precise timing values of detected AE peaks. These AE data are displayed as the Energy Rate (aJ/Hit) in all plots.

**Figure 2:**
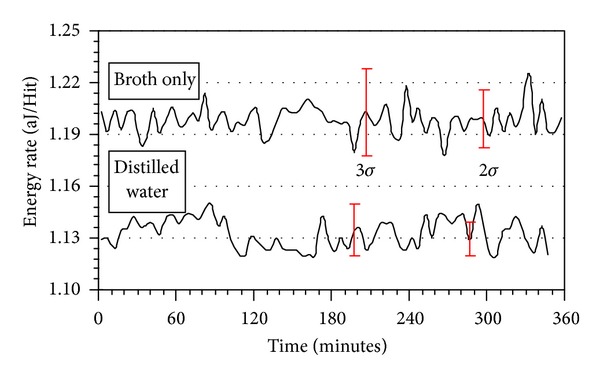
The AE patterns from growth cells containing nutrient broth or distilled water at 32°C with ±2*σ* and ±3*σ* deviations displayed.

A Gaussian distribution analysis was performed on all energy rate data. It provided ±*σ* standard deviations that gave a level of inherent and background noise contributions to the AE signals and was used as the determinant for whether bacteria-containing broths before and after exposure to the metabolic inhibitor or c2 Bacteriophage had intensities beyond noise variations. The basis for the analyses was an assessment of AE data from nutrient broths without bacteria or from de-ionized water, accumulated over periods typical for the bacteria growth cycles.  Figure 2 shows AE data and corresponding ±2*σ* and ±3*σ* deviation bars for these null tests. A ±3*σ* deviation was sufficient to encompass all the variations in the AE signals whereas a ±2*σ* deviation was not sufficient. Hence, all AE data were assessed relative to a ±3*σ* standard. Intensities above this level were considered significant and related to the presence of microbial activity; intensities below ±3*σ* were considered insignificant and are not discussed below.

**Figure 3:**
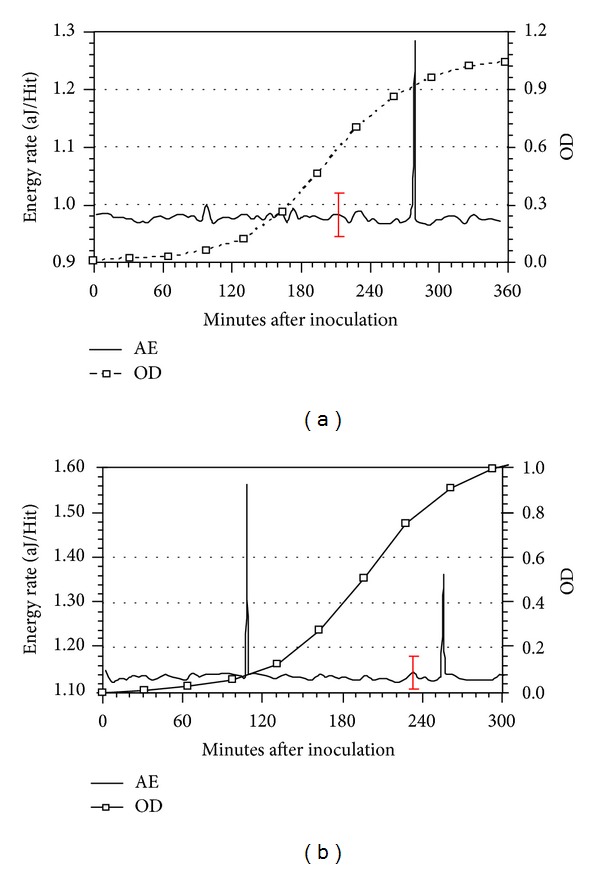
(a) OD and AE data acquired during growth of *L. lactis* at 32°C with initial amount 10^3^ cfu/mL (±3*σ* deviation displayed). (b) OD and AE data acquired during growth of *L. lactis* at 32°C with initial amount 10^6^ cfu/mL (±3*σ* deviation displayed).

**Figure 4:**
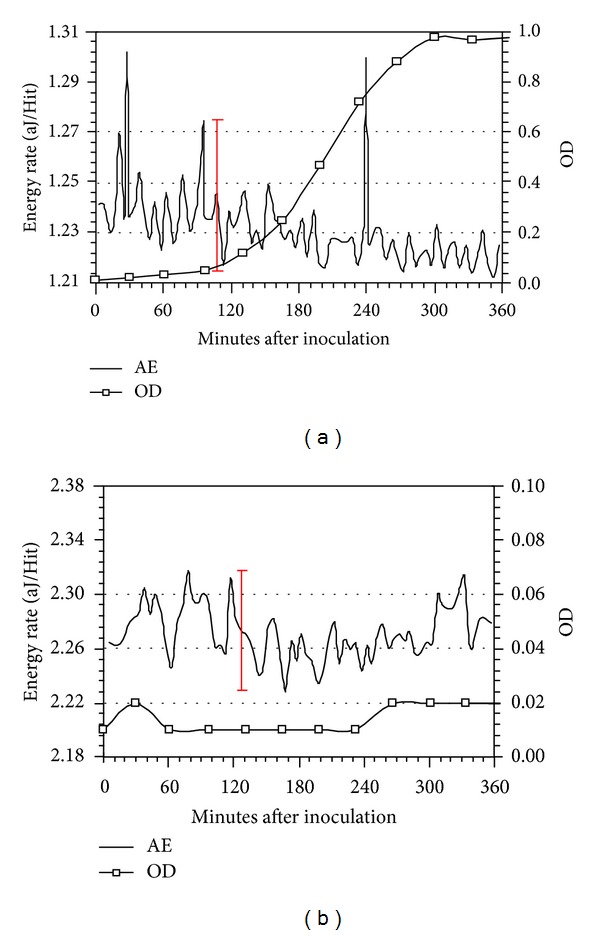
(a) OD and AE data acquired during growth of *L. lactis* at 32°C with initial amount 10^6^ cfu/mL (±3*σ* deviation displayed). (b) OD and AE data acquired during growth of *L. lactis* at 32°C with an initial amount of 10^6^ cfu/mL and with injection of NaN_3_ simultaneous with bacteria inoculation (±3*σ* deviation displayed).

OD data acquired from samples extracted from nutrient broths and the corresponding AE data during normal growth of *L. lactis* at 32°C, that is, without injection of metabolic inhibitor or c2 bacteriophage, are shown in Figures 3 and 4. The OD data show the traditional lag, log, and stationary growth phases of bacterial life cycles. The AE data represent one-minute averages of the absolute energy (in attojoules, i.e., 10^−18^ joules)-per-Hit and labeled as the Energy Rate, aJ/Hit. The plots also contain ±3*σ* deviation bars.

For an initial bacterial amount of 10^3^ cfu/mL, Figure 3(a), one AE peak having intensity beyond ±3*σ* was detected at 278.5 minutes; for bacterial amount of 10^6^ cfu/mL, Figures 3(b) and 4(a), two AE peaks having intensities beyond ±3*σ*, were detected at 108.5 and 255.5 minutes (Figure 3(b)) and at 27.5 and 239.5 minutes (Figure 4(a)).

The AE data from *L. lactis* during “normal” growth did not show a relationship between peak intensities and initial bacterial amounts. This absence is believed primarily to be a consequence of not having quantitative colony forming units (cfu) data with which to compare to AE data. The OD data are qualitative and follow changes in broth turbidity as the amount of bacteria increases whereas standard plate count methods [12] produce more precise information about cfu count. From other tests using the plate count method during *L. lactis* growth it is known that the cfu values increased by ~10 fold within 120 minutes after bacteria inoculation but the final stationary phase cfu values were 10^2^–10^4^ greater than the inoculation amounts depending on initial amount and other experimental procedures. In other words, it was possible that the actual bacterial amount during the late log phases were nearly identical for the OD data presented in Figure 3(a) versus Figures 3(b) and 4(a), even though initial amounts were significantly different.

A listing of AE peak having intensities greater than ±3*σ* and proposed to be associated with *L. lactis* activity are given in Table 1 for growth conditions labeled “normal”, and “with phage” injection (AE and OD plots acquired after bacteriophage addition are presented hereafter). The AE peaks detected were either when OD values <0.3 or when OD values >0.75. No AE peaks were observed beyond 280 minutes after bacteria inoculation, that is, when OD values were >0.90.

Hence, AE peak timings under normal growth conditions were either in the early-log or late-log phases of growth. Detection of AE activity during the early log phase, that is, ≤150 minutes after inoculation, is considered consistent with the onset of cellular or physiologic events that serve as the starting point of this phase, including chromosome duplication, DNA replication, and a substantial increase in metabolic rate. Furthermore, the pattern of bacterial growth at the beginning of the log phase is a burst in the cell population as duplication begins via binary fission. Near the end of log phase (~280 minutes after inoculation), the cell population undergoes a similar burst in numbers before entering a more limiting or metabolically-controlled environment. This burst ensures the production of a maximum number of progeny cell within the remaining available nutrient supply. In contrast, there is minimum cell division during the lag phase, where cell activity is associated with cellular and/or organismal adaptation to the new physiological and metabolic environment sensed by the individual bacterial cells. During the lag phase, cells may increase in size due to a change in their metabolic activities, but they do not divide by binary fission [13–15].

To further identify that AE peaks were related to bacteria activity, tests were performed in which *L. lactis* was inoculated at 10^3^–10^6^ cfu/mL and simultaneously NaN_3_ was added into the growth media.  Figure 4(b) summarizes results from these tests: the OD data depict complete metabolic inhibition, that is, no increase in bacterial amounts, and the AE data show no peaks with intensities beyond ±3*σ*. Therefore, the acquisition of AE data was able to distinguish the presence of *L. lactis* metabolic activity.

### 3.2. c2 Bacteriophage Infection

#### 3.2.1. OD Data, Phage Infection

The OD and AE data were acquired during *L. lactis* growth before and after addition of Bacteriophage c2 to determine whether characteristic AE was produced as a consequence of the infection. To increase clarity of the ensuing discussion, the AE data are divided into three comparative sections defined by the m.o.i level of the phage infection. Levels were arbitrarily labeled as Low, Medium, and High as defined by OD values that were attained 300 minutes after phage infection.

**Figure 5:**
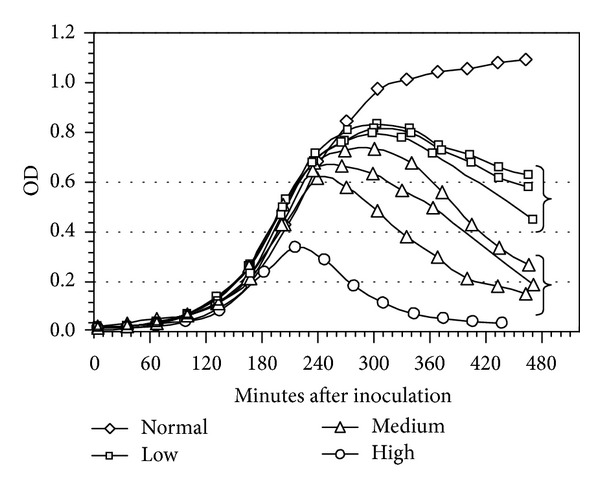
Optical density data obtained during *L. lactis* during normal growth at 32°C and after c2 phage addition for low, medium, and high m.o.i levels.

Figure 5 shows the OD curves obtained from the phage infected cultures in comparison to the OD curve from a normal growth test. At ~300 minutes after c2 injection, low m.o.i OD values were between 0.45–0.63; medium m.o.i OD values were between 0.15–0.27 and high m.o.i OD values were ~0.03. The low and medium m.o.i data were acquired when c2 was added to the broth 165 minutes after bacteria inoculation; the high m.o.i data were acquired when c2 was added to the broth 130 minutes after bacteria inoculation.

The m.o.i level could be changed because the efficacy of phage-initiated cell lysis was dependent on the availability of Ca within the nutrient broth; this effect is well known [16, 17]. Without Ca, phage-initiated lysis of *L. lactis* did not occur; depending on when a 0.01% Ca solution (from CaCl_2_) was added to the broth, lysis rates were found to vary over a wide range as defined by the OD data. Absence of lysis may be related to a calcium mediated signal transduction mechanism for phage-bacteria attachment, in which the initial phase requires a signaling cascade mediated by the presence of calcium [18]. Once the cascade is initiated, the process begins for viral protein attachment to bacterial glycoprotein receptor by alteration of protein structure. Hence, when c2 was to be added to the culture 165 minutes after bacteria inoculation the addition of CaCl_2_ to the broth was accomplished either at the time of c2 addition or 30 minutes before its addition. In this manner it was possible to produce OD curves in which the deleterious effect of c2 was differentially amplified and, as a consequence, gave low or medium phage m.o.i levels.

Timing information about phage infection was developed from the OD data and is presented in Table 2. Included are timings of infection after bacteria inoculation; the first indication when OD values from the infected cultures were less than OD values from “normal” growth tests-labeled as “1st OD change” in the table; the maximum OD values; differences between the (1st OD change − phage infection) and between the (Maximum OD − phage infection).

Phage addition timings for low and medium m.o.i were identical at 165 minutes; the subsequent timings of the 1st OD change were also approximately constant between 218–235 minutes. The timings of the maximum OD values were greater for low m.o.i in comparison to medium m.o.i; although, there was greater variability for medium m.o.i than for low m.o.i, a trend that followed a greater variability in overall OD values from the medium m.o.i tests (Figure 5).

Values of the 1st OD decrease were determined by comparing the shape of the OD plots of “normal” growth relative to those of phage infected growth and then by extrapolating between OD sampling times that were accomplished approximately every 30 minutes. The (1st OD decrease − infection) differences varied between ~50–70 minutes, with low m.o.i tests displaying the largest values. The smallest value—47 minutes for high m.o.i—determines an upper limit of the period for overall phage infection, replication and cell lysis when phage addition was accomplished during the early part of the bacteria log growth phase, whereas the largest values—70 minutes—determine an upper limit of this period when phage addition was accomplished during the mid-to-late part of the bacteria log growth phase. During these periods it would be necessary for phage to diffuse to bacteria cells; adsorb and irreversibly attach onto cells; inject their genome; replicate within the cells; eventually cause cell lysis.

#### 3.2.2. AE Data, Phage Infection

In the following only AE peaks with timings equal or greater than the respective phage addition time are discussed; it is assumed that AE peaks detected before c2 addition were associated with bacteria activity and are listed in Table 1 under the category “with phage”.

**Figure 6:**
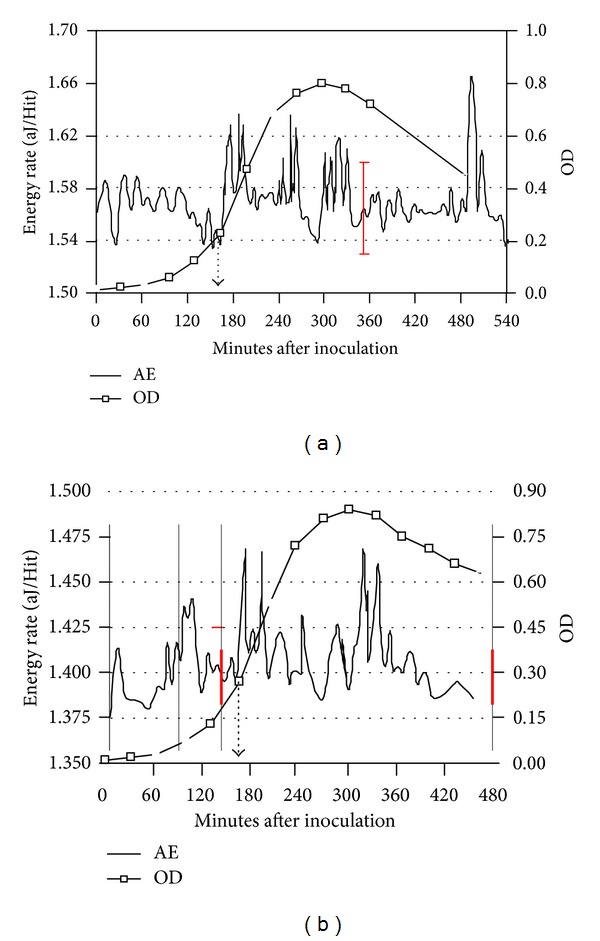
(a) and (b) AE and OD data from *L. lactis* at 32°C before and after c2 Bacteriophage infection 165 minutes after bacteria inoculation; phage addition time is indicated by dashed arrow. (Low phage m.o.i.).

For low phage m.o.i, the OD and AE data in Figures 6(a) and 6(b) are presented for two different tests in which a *L. lactis* culture was inoculated at time = 0, and then the c2 was added 165 minutes later. The highest OD values for the c2-infected cultures were near 0.85, and occurred ~135 minutes after the addition of c2.

The AE data in Figure 6(a) contain groups of peaks with intensities greater than ±3*σ* at [177.5, 188.5 and 193.5], [245.5, 255.5 and 262.5], and [300.5, 310.5, 320.5 and 330.5] minutes after inoculation. Although the groups near 180 and 250 minutes could be associated with bacterial activity, because *L. lactis* under “normal” growth produced AE peaks as great as 278 minutes after inoculation (Table 1), the AE data from “normal” growth did not have the complexity nor multiplicity of peaks shown in Figures 6(a) and 6(b). In these latter two figures, AE peaks having timings greater than 278 minutes are assigned to be phage-related because *L. lactis* under “normal” growth produced no AE peaks beyond 280 minutes.  Table 3 lists all of the AE peaks having values greater than the phage infection times for the low, medium and high m.o.i tests.

The AE data in Figure 6(b) contain groups of peaks with intensities greater than ±3*σ* at [173.5 and 193.5], [244.5 and 293.5], and [320.5 and 337.5] minutes after inoculation. The phage-related AE signals are assigned to the 293.5, 320.5 and 337.5 minute peaks.

**Figure 7:**
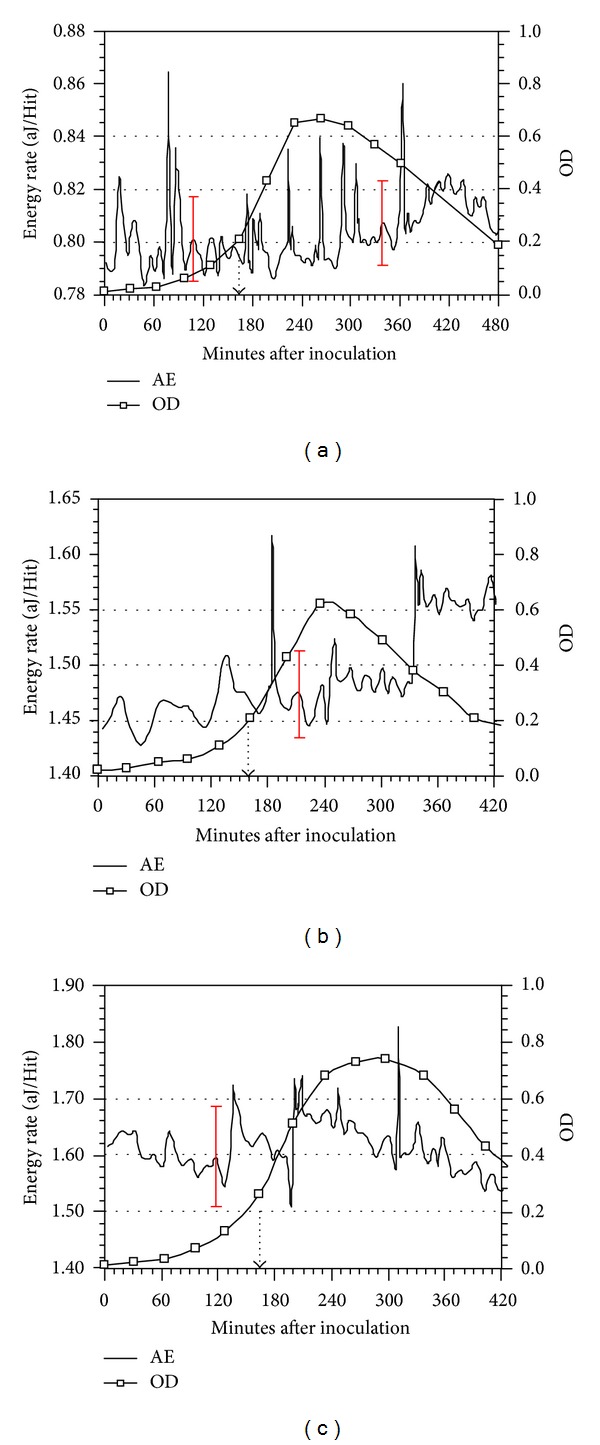
(a), (b), and (c) AE and OD data from *L. lactis* at 32°C before and after c2 bacteriophage infection 165 minutes after bacteria inoculation; phage addition time is indicated by dashed arrow. (Medium phage m.o.i).

For medium m.o.i of phage, the OD and AE data in Figures 7(a), 7(b), and 7(c) are presented in which c2 Bacteriophage was also added 165 minutes after inoculation of *L. lactis*. The AE data in Figure 7(a) contain peaks with intensities greater than ±3*σ* at 173.5, [223.5, 262.5 and 290.5], 306.5, and 363.5 minutes after inoculation. The AE data in Figure 7(b) contain peaks with intensities greater than ±3*σ* at 188.5, 237.5, and 301.5 minutes; in Figure 7(c) the peaks are at 201.5, 208.5, 247.5, and 310.5 minutes. The AE peaks with timings greater than 280 minutes are assigned to be phage-related.

**Figure 8:**
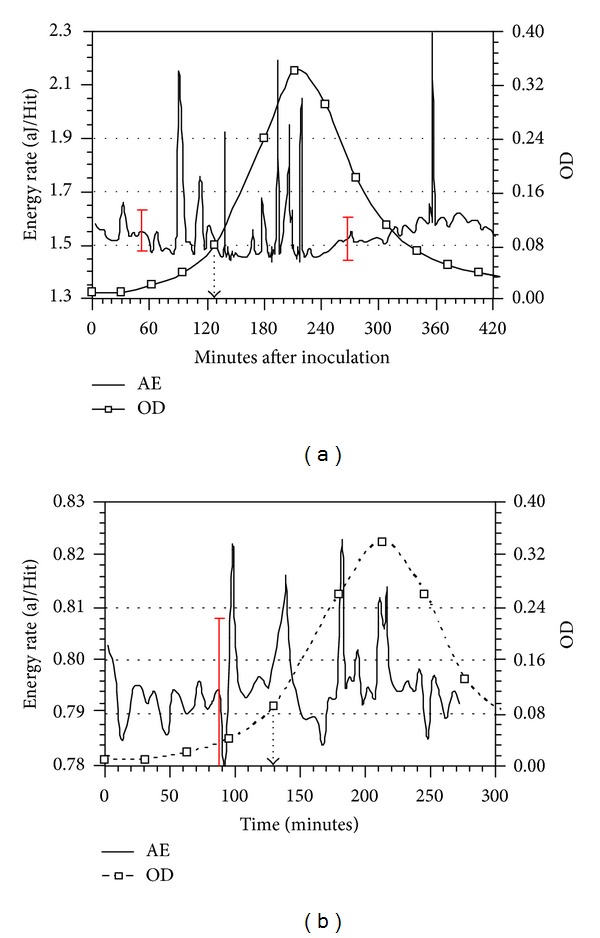
(a) and (b) OD and AE data acquired during growth of *L. lactis* with an initial amount of 10^6^ cfu/mL and injection of c2 Bacteriophage 130 minutes after bacteria inoculation; phage addition time is indicated by dashed arrow. (High phage m.o.i).

For high m.o.i of phage, the OD and AE plots in Figures 8(a) and 8(b) present data for two different tests in which a *L. lactis* culture was inoculated at time = 0, and then the c2 addition was accomplished 130 minutes later. The AE peaks having timing beyond the phage addition and intensities greater than ±3*σ* in Figure 8(a) are 138.5, [177.5, 194.5, 205.5, and 219.5], and 356.5 minutes; in Figure 8(b) the AE peaks are at 138.5, 182.5, 211.5, and 216.5 minutes after inoculation. Only the 356.5 minute peak timing in Figure 8(a) is beyond those detected under “normal” growth conditions.

Table 3 presents a list of the detected AE peaks having timings after the phage addition along with some corresponding OD data. In general, significant groups of peak timings are easily noted: (a) within ~30 minutes after phage addition, that is, between ~180–210 minutes; (b) near 250; (c) near ~320 minutes.

Energy rates of each of the peaks were compared to baseline energy rates of each test to define in more detail the phage-related activities that could cause AE and whether they were associated with bacteria or phage processes. Intensities of the peaks above their baseline values were calculated as a percentage increase, with the following outcome as related to Phage m.o.i: Low/Medium/High = 7.6%/11.0%/29.1%. Hence, higher fecundities were associated with higher AE peak intensities. This association would not be expected if the acoustic signals originated from bacteria metabolic activity because higher phage fecundities force less metabolic activity: therefore, all AE peaks listed in Table 3 are proposed to be associated with processes associated either with the decrease in bacterial amounts or increase in phage amounts. Two possible biophysical processes that could conceptually produce detectable AE are the lysis of the bacterial cells and the injection of phage genome into the cells.

Upon maturation within the bacteria cells, the lysis of *L. lactis* cells by c2 could be detected if the Hit energies were ~1 aJ or greater and the intensity losses at the interfaces between the sensors and the nutrient broth did not eliminate too much intensity. Similarly, after phage attachment to bacteria cell walls, the injection of phage nucleic acid could also be detected if the energetics and dynamics were great enough. Although concise data about the energy and dynamics of *L. lactis* lysis are not known, an estimate of the mechanical energy associated with phage nucleic acid injection into bacterial cells can be calculated by knowing forces near 50 pN are exerted by motor proteins in phage capsids over their ~25 nm radius [19]. Hence, the energy of nucleic acid injection could be as great as 1250 aJ = 50 pN × 25 nm, a value far in excess of that detectable by using piezoelectric sensors.

Nearly all lactococcal phage species conform to one of two morphotypes: B1, having a small isometric head and a long noncontractile tail; and B2, having a prolate head and a long noncontractile tail [18]. Tailed phages usually lyse their host cells in a rapid sequential process by an enzyme called endolysin, which attacks and breaks down the cell wall peptidoglycan. In the case of a multiplicity of infection (MOI) of ≥100, cell lysis rates are typically rapid, and when the infected cells lyse the progeny infection phage can find it more difficult to infect other cells if a rapidly declining bacterial cell count was caused by the infection. In contrast, slow lysis rates become evident at low MOI's near 5 [20]. Thereby, phage m.o.i is affected by the dynamics of the bacterial cell count and the ratio of phage-to-bacteria, but it can also be affected by when in the bacteria growth curve the phage was introduced. During the tests described herein the MOI's are estimated to be ~10 at the time of phage addition.

Therefore, it was assumed that AE peak intensities would be dependent on either cell lysis after phage intracellular replication or phage genome injection. One approach used to assess these two possibilities was to examine OD timings relative to AE peak timings. The first AE peaks were between 172.5–193.5 minutes for low m.o.i, 173.5–201.5 minutes for medium m.o.i, and at 138.5 minutes for high m.o.i. Except for Test #3 with medium m.o.i, all 1st AE peak values were less than 30 minutes after phage addition to the nutrient broth. If these peaks were associated with cell lysis, then it would be necessary upon c2 addition for the phage to diffuse to the cells, attach and inject nucleic acid, and then replicate and cause lysis in less than 30 minutes after phage addition into the broth. Because it has been shown that c2 bacteriophage latent periods are between ~30–85 minutes [21, 22], values which do not take into account diffusion of the phage to the bacterial cells, the initial AE peaks after phage injection could not have been caused by cell lysis.

Then, the timings of the 2nd AE peaks in Table 3 were assessed relative to the OD timings. For low m.o.i these peaks were between 237.5–262.5 minutes, all values of which were greater than the value of the 1st OD change shown in Table 3. For high m.o.i the aforementioned peaks were between 237.5–262.5 minutes, all values of which were greater than the value of the 1st OD change shown in Table 3. Hence, for low and high m.o.i cell lysis could not have caused the 2nd AE peaks. However, this conclusion is not as clear for all of the medium m.o.i tests because one of the three tests (Test #1) had the 2nd AE peak value greater than the 1st OD change value, whereas the other two tests did not.

It was also discovered that the full width half maximum (FWHM) values of all AE peaks with timings after phage addition were small (~1.6 minutes). Their analyses required careful attention to avoid their removal by signal averaging when periods of three minutes or greater were used. In contrast, AE peaks associated with bacteria activity displayed in Figures 3(a)–4(a) were unaffected by five minute signal averaging. Sensitivity to signal averaging implies that short duration (~1 minute) acoustic events were detected after phage addition. The process leading up to genome injection is a highly regulated two-step process requiring coordination of biophysical processes, such as pressure within viral capsids, with molecular processes, such as translation of viral proteins. The formation or breakage of bonds during these molecular processes could lead to energy releases between ~10–150 aJ [23] during cascading reactions. After the stage is set, the timescale of genome injection is expected to be short with a corresponding energy which could be an order of magnitude greater [19] than bond formation energy. In contrast, cell lysis is a multistep, complex process also requiring coordination of biophysical and molecular processes that includes the action of the cell wall degrading lysine enzyme which eventually creates a hole in the peptidoglycan layer of the bacteria cell membrane through which progeny virions begin to be released and, then, the cell membrane disintegrates. The differences in dynamics between genome injection versus cell lysis may be expected to create distinctly different acoustic signals, one aspect of which is a shorter duration but more energy intensive signal during genome injection.

Table 3 also presents time differences between peaks for each test, labeled A-PA, B-A, and C-B values of which are used to give additional clarification of the AE data. First, it is assumed that the peak groupings in columns labeled as A, B, and C represent timings of primary, secondary, and tertiary c2 genome injection cycles. The durations between the first injection cycle and phage addition, labeled as PA-A in the table, were the smallest (8.5 minutes) for high m.o.i, and on average were approximately equal (~21 minutes) for medium and low m.o.i tests. If B-A values represent the time required between primary and secondary genome injection cycles, then the duration includes the timings of phage genome injection, genome replication, cell lysis, diffusion of progeny virions to cells, and subsequent viral transcription and irreversible adsorption onto cells. On average, B-A durations were the smallest (41.5 minutes) for high m.o.i, intermediate (54 minutes) for medium m.o.i, and the greatest (69.7 minutes) for low m.o.i. The C-B cycle would require the same phage and cellular processes to occur as for B-A durations; the C-B durations on average were smallest (59.5 minutes) for low m.o.i, intermediate (83.2 minutes) for medium m.o.i, the greatest (179 minutes) for high m.o.i.

Durations of overall phage replication cycles are affected by four dependent variables: nutrient concentration; free or un-infected bacterial cell amount; free phage amount; and infected bacterial cell amount [24]. For a primary infection cycle it has been shown that the period between phage introduction and *L. lactis* cell infection would be near 30 minutes [22]. This value, greater than all A-PA values except for Test #3, medium m.o.i in Table 3, would be influenced by these phage, bacteria and nutrient variables as would the other cycles. Because of the difference in phage addition times (130 minutes, high m.o.i versus 165 minutes, low and medium m.o.i) the phage-to-bacteria ratios would have been greater (~10) upon phage addition for high m.o.i tests than for low or medium m.o.i tests. This difference in phage-to-bacteria ratios would have occurred also for the 2nd and 3rd genome injection cycles, but after the 2nd cycle the free bacteria amount was so highly depleted during high m.o.i tests that the timing of the 3rd cycle was greatly increased relative to the low and medium m.o.i levels. For medium m.o.i the timing of the 3rd cycle also increased relative to the 2nd cycle, but not to the extent of high m.o.i, whereas because of a remaining high amount of free bacteria during low m.o.i tests the timing of the 3rd cycle decreased relative to the 2nd cycle. Therefore, timing values of the proposed cycles in Table 3 follow expectations for the AE peaks to be caused by phage genome injection.

The presence in Table 3 of multiple AE peaks in groups with timing differences as small as five minutes implies distinct and coordinated genome injection processes. Although not discussed herein, it is also possible that each of the groups in each column of Table 3 represent multiple groups. For example, in the 2nd AE peaks listing for Test #2, medium m.o.i the time differences are (262.5 − 223.5 = 39 minutes; 290.5 − 262.5 = 28 minutes) and for Test #1, high m.o.i the time differences are (205.5 − 177.5 = 28 minutes; 219.5 − 194.5 = 25 minutes), values of which are close to a published c2 latent period [22].

Furthermore, virus burst sizes are also known to affect the rate of cell lysis and phage-to-bacteria amount ratios [25–27]. Intensities of AE peaks caused by phage genome injection would be expected to be impacted by burst sizes but data on burst sizes were not acquired. Further testing could shed light on this possibility.

The data presented herein were analyzed after completion of each test. However, the detection of phage genome injection into bacterial cells using AE techniques has the potential to be accomplished in real-time. This capacity would expand overall applicability of the AE technique for probing virus-cell interaction dynamics and for shedding insight into the prevention of viral infections. Additionally, the technique will serve as a novel way to study and analyze the critical steps of pathogenic life cycle in general, including the molecular basis of infection, the defensive mechanism by the host cell as well as host-pathogen interaction. Also, improved or new sensor-cell attachment mechanisms that optimize contact between the surfaces or immerse the sensor within nutrient media would help circumvent the AE transmittance losses at interfaces; for example, the loss of AE signal intensity at the broth-glass interface combined with at the glass-stainless steel sensor interface eliminated more than 75% of the intensity from the source of the acoustic wave. Nevertheless, the data presented herein show that AE signals from bacteria metabolic activity and phage genome injection were measurable and distinguishable, and the creation of a metabolically quiescent culture caused elimination of AE from the bacteria.

## 4. Conclusions

It was discovered that the presence or absence of metabolic activity by bacteria *Lactococcus lactis* ssp. *lactis* C2 could be detected by measuring acoustic signals from cultures using piezoelectric sensing elements attached to the outside of bacteria growth flasks. The bacteria-related AE signals were evident when the bacterial amount attained ≥10^4^ cfu/mL levels and only occurred during early or late periods of the logarithmic growth phase; these timings suggested a relationship between cell division and measurable acoustic emission, consistent with the higher metabolic activities during the steps of bacterial life cycle. It was also discovered that Bacteriophage c2 infection of *L. lactis* could be detected, and the origin of the ensuing AE signals was proposed to be a result of phage genome injection into the bacteria cells. This data has an important biological significance in terms of the real time study of phage mediated infection cycle and its consequences in the phage as well as host bacterium, at both molecular and cellular level. The multiplicity of the AE signals detected over ~250 minutes after phage addition to the bacteria culture contained timings that were in agreement with previous published c2 data during the infection of *L. lactis* and with the dynamics expected from virus-microbe assemblages under rapidly changing amounts. This multiplicity also suggested-extensive coordination of the timings of phage genome injection into bacteria cells. The acoustic detection of microbes could serve as a potentially valuable and real-time approach to address numerous basic and applied biological and/or clinical issues related to pathogenic microbial infections.

## Figures and Tables

**Table 1:** The timing of AE peaks with greater than ±3*σ*; intensities that are related to bacteria activity during *L. lactis *growth at 32°C.

Test Description (PM = phage m.o.i)	cfu/mL at inoculation	Minutes after inoculation	Minutes after inoculation	Minutes after inoculation
Normal growth	10^3^			278.5
Normal growth	10^3^			262.5
Normal growth	10^3^		172.5	

Normal growth	10^6^		182.5	
Normal growth	10^6^	27.5		239.5
Normal growth	10^6^	32.5	142.5	
Normal growth	10^6^		108.5	255.5
Normal growth	10^6^			262.5

With phage, High PM	10^7^	90.5, 98.5	112.5	
With phage, Medium PM	10^6^	88.5		
With phage, Low PM	10^6^	86.5	107.5	

**Table 2:** Optical density (OD) data acquired during *L. lactis *growth and infection with c2 Bacteriophage showing times for the phage addition; 1st OD change; maximum OD values; difference between the 1st OD change and addition of phage (1st OD change − addition); difference between the Maximum OD and addition of phage (Maximum OD − addition).

	Low m.o.i			Medium m.o.i			High m.o.i		
	Timing, minutes			Timing, minutes			Timing, minutes		
Phage addition	165	165	165	162	165	165	128
1st OD change	225	225	235	218	235	225	175
Maximum OD	297	297	302	236	264	286	213
1st OD change − addition	60	60	70	56	70	60	47
Maximum OD − addition	132	132	137	74	99	121	85

**Table 3:** Summary of AE peaks (in minutes) detected during the infection of *L. lactis* with Bacteriophage c2 with 10^9^ pfu/mL at 32°C. The 1st AE cycle, 2nd AE cycle, and 3rd AE cycle labeling represent proposed primary, secondary, and tertiary sequences of phage-related AE signals.

m.o.i Level	Test #/1st OD change	Phage addition PA	1st AE peaks A	2nd AE peaks B	3rd AE peaks C	A-PA	B-A	C-B
Low	1/225	165	177.5	245.5	300.5	12.5	68	55
			188.5	255.5	310.5	23.5	67	55
			193.5	262.5	320.5	28.5	69	58
					330.5			
	2/235	165	173.5	244.5	319.5	8.5	71	75
			193.5	287.5	337.5	28.5	94	50
	3/225	165	188.5	237.5	301.5	23.5	49	64

Medium	1/218	165	184.5	250.5	336.5	19.5	66	86
					342.5			
	2/235	165	173.5	223.5	306.5	8.5	50	83
				262.5	363.5			101
				290.5				
	3/225	165	201.5	247.5	310.5	36.5	46	63
			208.5					

High	1/175	130	138.5	177.5	356.5	8.5	39	179
				194.5				
				205.5				
				219.5				
			138.5	182.5		8.5	44	
	2/175	130		211.5				
				216.5				
